# A Case of Alveolar Rhabdomyosarcoma of the Nasal Cavity in an Adult: An Unusual Location

**DOI:** 10.7759/cureus.61815

**Published:** 2024-06-06

**Authors:** Hajar El Bakouri, Oumayma Mezouari, Wafaa Merssetti, Mariame Harrak, Niama Ghozali, Hamza Zerbani, Nabila Sellal, Mohamed Elhfid

**Affiliations:** 1 Radiation Therapy, University Hospital of Tangier, Tangier, MAR; 2 Oncology, University Hospital of Tangier, Tangier, MAR

**Keywords:** treatment, radiation, nasal cavity, rhabdomyosarcoma, alveolar

## Abstract

Rhabdomyosarcoma is a common soft tissue tumor in children but rare in adults. Alveolar rhabdomyosarcoma represents a subtype of rhabdomyosarcoma, extremely rare in adults, especially within the nasal cavities. Therapeutic protocols for adults are often based on those used in pediatric cases.

We present the case of a 56-year-old female patient with a history of breast cancer who developed alveolar rhabdomyosarcoma of the nasal cavity, stage III, managed initially with chemotherapy resulting in partial response. Subsequently, the patient underwent concomitant chemoradiotherapy. The clinical course was marked by local remission with metastatic progression after 18 months.

Alveolar rhabdomyosarcoma is uncommon in adults, and its therapeutic management remains non-standardized. However, it is typically based on initial chemotherapy followed by local treatment.

Despite therapeutic advances, the prognosis remains poor.

## Introduction

Alveolar rhabdomyosarcoma is a high-grade, malignant, primitive neoplasm characterized by round cells and partial differentiation of skeletal muscles [1]; this can result in misdiagnosis and subsequently inappropriate treatment. Alveolar rhabdomyosarcoma of the nasal cavity or paranasal sinuses is rare, with most reported cases occurring in children [1,2]. The sites of predilection for rhabdomyosarcoma differ between children and adults [3]. In children and adolescents with rhabdomyosarcoma, the genitourinary tract is the most common primary site (21%). Rhabdomyosarcoma represents only 3% of all soft tissue sarcomas in adults [4]. Alveolar rhabdomyosarcoma is rare at any age, particularly when it involves the nasal cavity, with only a few cases documented in the literature [5].

Our case report aims to describe the clinical characteristics and prognostic factors of rhabdomyosarcoma of the nasal cavity in adults.

## Case presentation

Our patient is a 56-year-old female, treated in 2016 for breast cancer with surgery followed by radiation and hormonal therapy, with no history of smoking or alcohol drinking. She had a family history, including a daughter currently undergoing treatment for stomach cancer and a paternal aunt who died from metastatic liver cancer at the age of 51. She has not undergone genetic testing.

**Figure 1:**
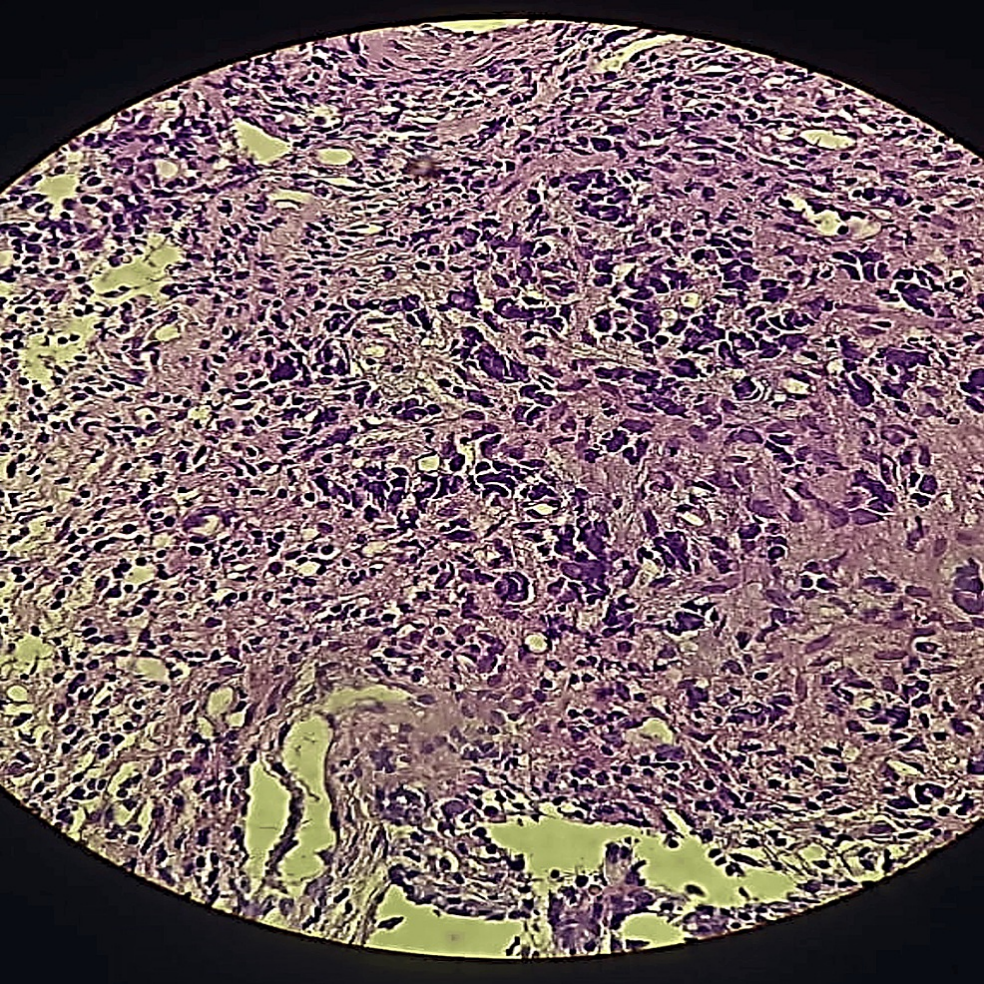
Histopathological images (H&E stain) x40, showing a mucosa composed of a regular respiratory-type epithelium and a stroma largely occupied by a malignant tumor proliferation of pleomorphic round cells arranged in diffuse sheets.

In February 2022, she presented with unilateral right nasal obstruction and episodes of mild ipsilateral epistaxis. A biopsy of the nasal cavity was performed. Microscopic examination revealed nasal mucosa lined by ciliated columnar epithelium with regular nuclei and a malignant tumor proliferation composed of variably sized cells with reduced eosinophilic cytoplasm, lacking mucosecretory features, and with indistinct cytoplasmic borders. These cells exhibited large, rounded, hyperchromatic nuclei with mitotic figures. The cells were arranged in variably sized sheets within a thin fibrous stroma (Figure 1).

**Figure 2:**
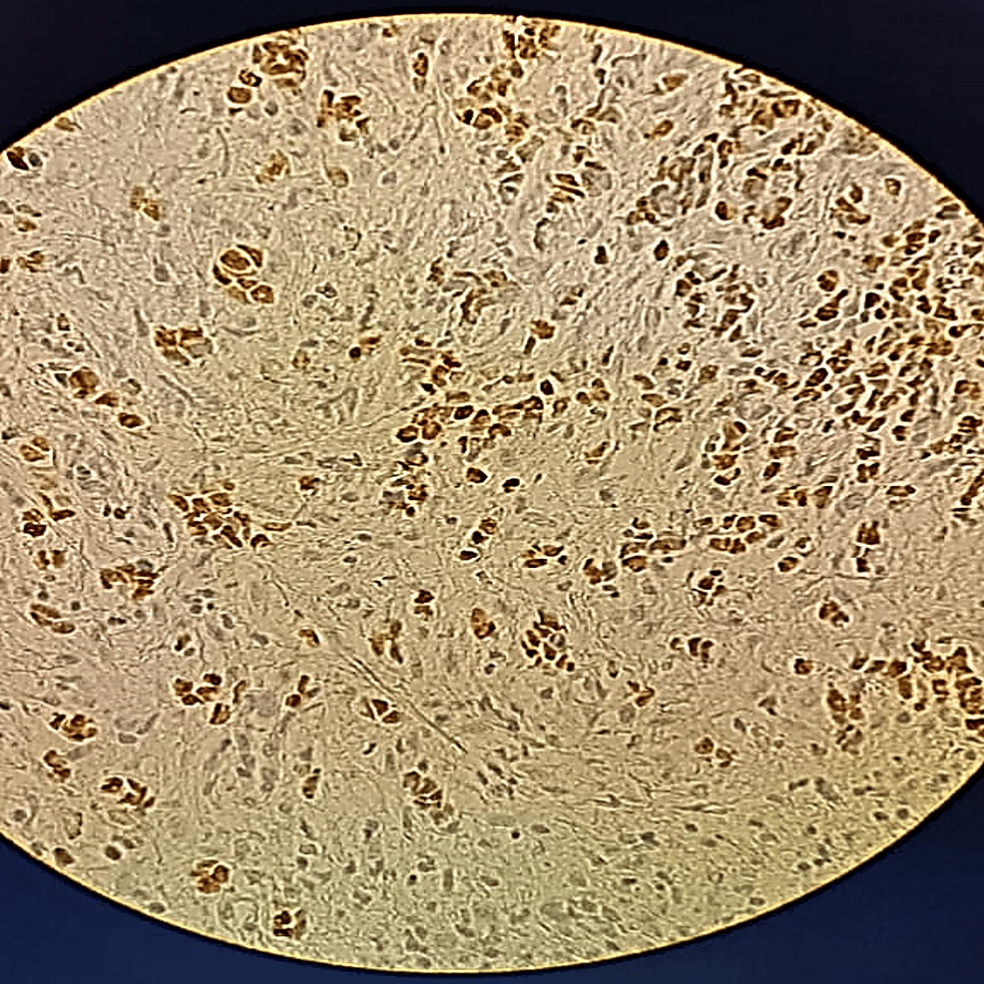
Immunohistochemical staining images x40 Myogenin: diffuse, strongly intense nuclear staining

**Figure 3:**
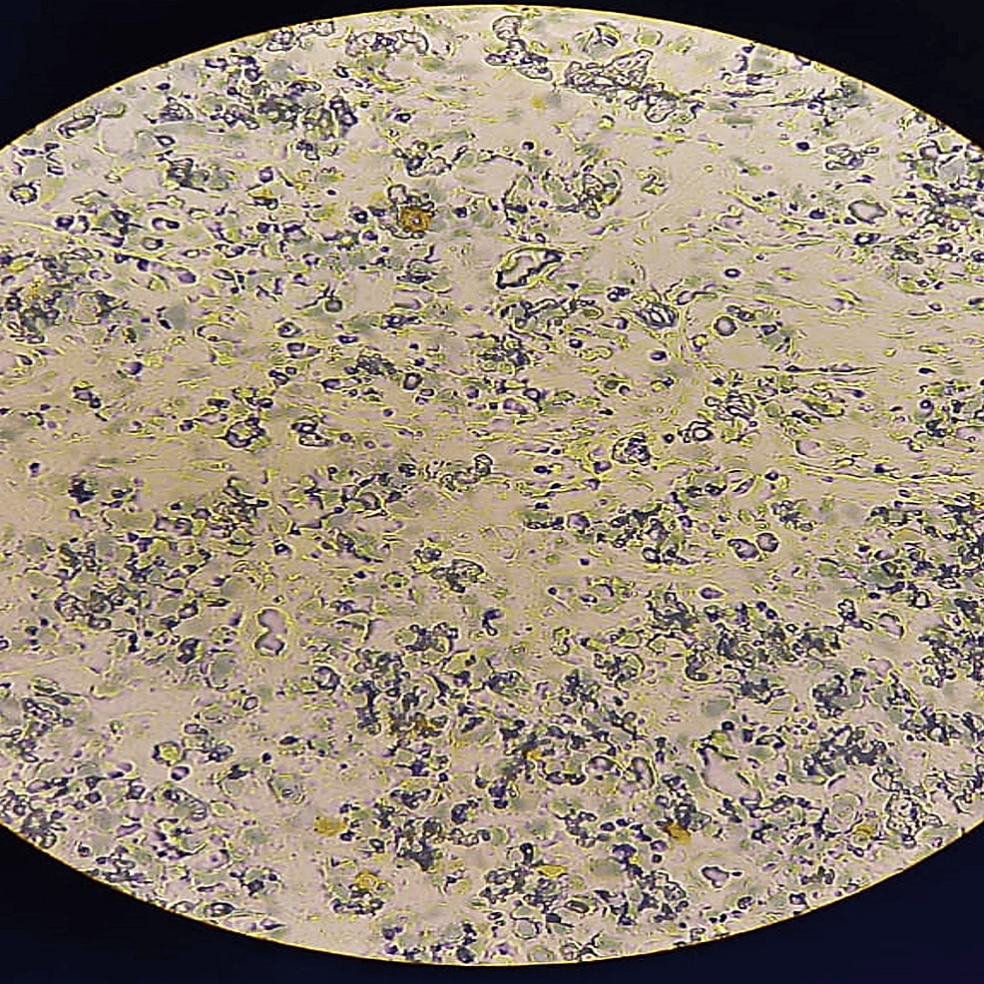
Immunohistochemical staining images x40 Desmin: multifocal, positive cytoplasmic staining

An immunohistochemical study showed that the tumor cells were negative for cytokeratins, S-100, P63, CD20, CD30, synaptophysin, and chromogranin A, but positive for desmin and anti-myogenin antibodies, which are highly specific and sensitive for alveolar rhabdomyosarcoma (Figure 2, Figure 3).

**Figure 4:**
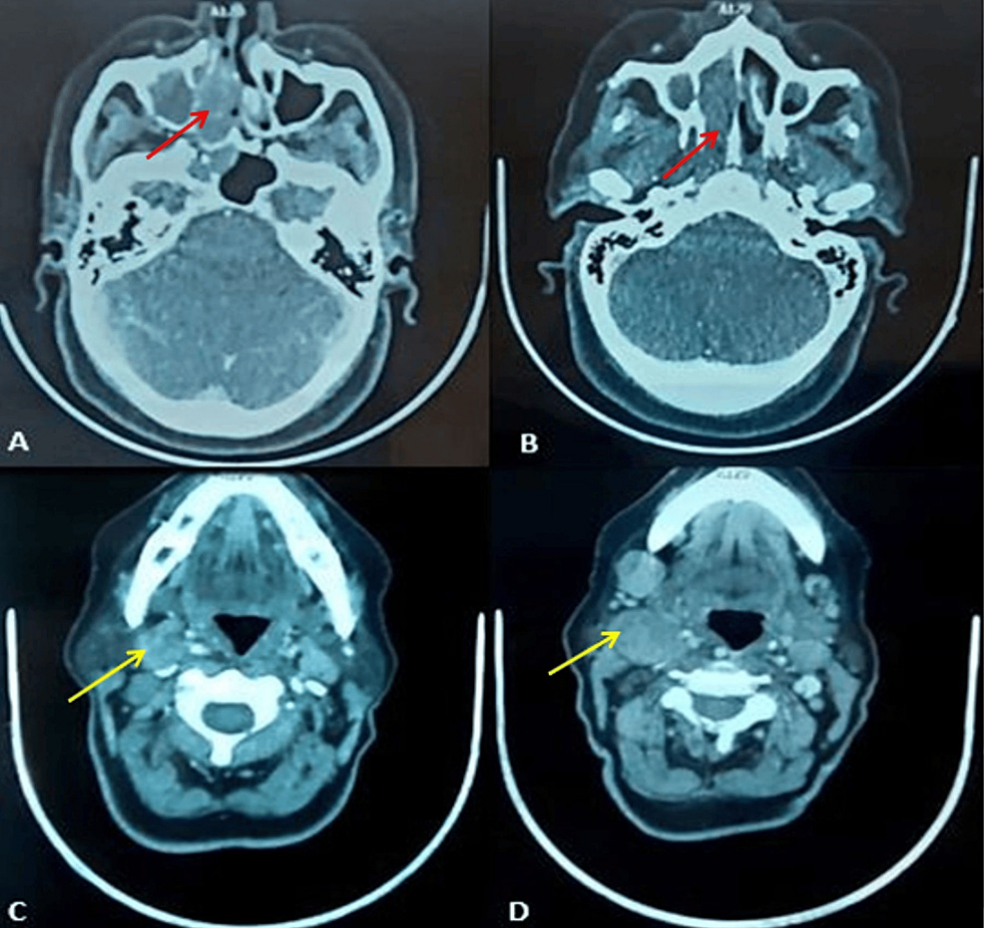
Computed tomography scan images A, B: Axial view showing a mass in the right nasal cavity with lysis of the nasal septum (red arrow). C, D: Axial view showing cervical lymphadenopathy (yellow arrow)

The patient underwent a cervicofacial computed tomography scan, which indicated a mass in the right nasal cavity with the erosion of the nasal septum and medial wall of the right maxillary sinus with intra-sinus extension, extending to the sphenoid sinus and surpassing the inferior turbinate reaching the right nostril, with bilateral submandibular lymphadenopathy, the largest on the right measuring 32x30 mm (Figure 4). A subsequent thoraco-abdomino-pelvic CT scan revealed no distant secondary localizations.

The clinical stage of the disease before starting therapy was unfavorable Stage III. To reduce the tumor size before surgery, the patient received neo-adjuvant chemotherapy based on epirubicin and doxorubicin.

**Figure 5:**
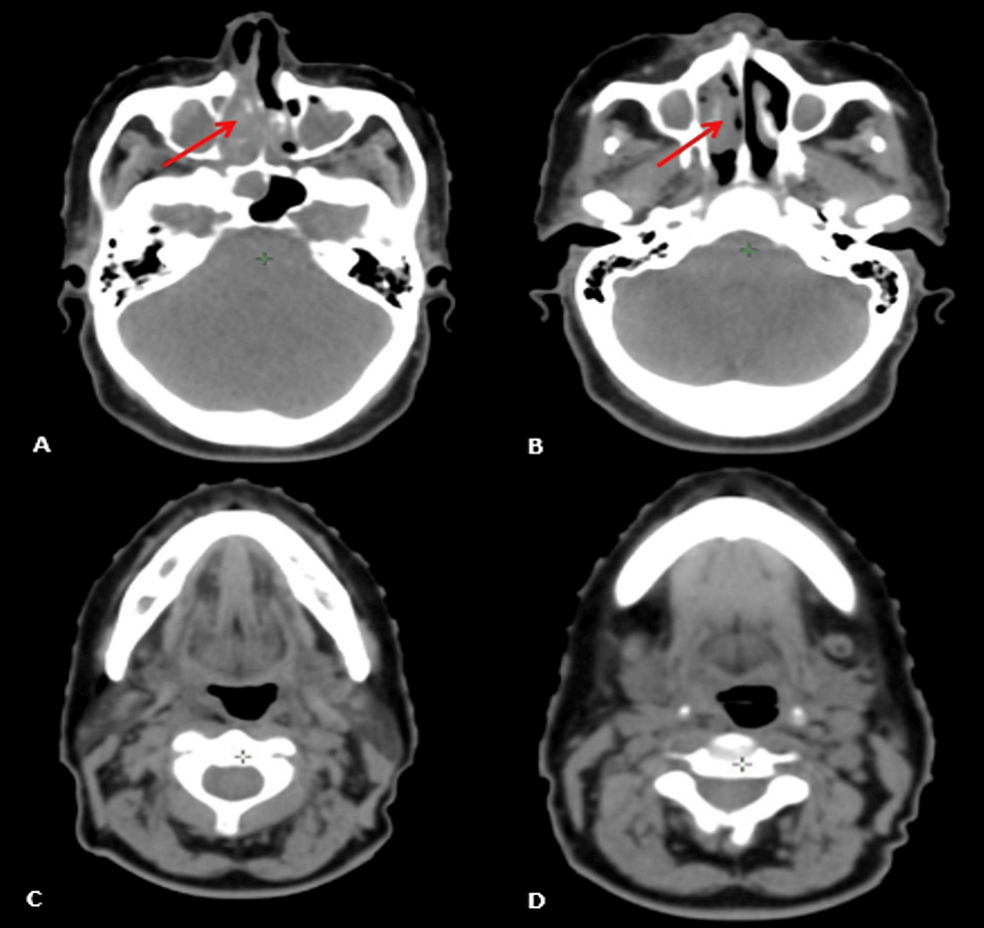
Computed tomography scan images Post-chemotherapy scan images A, B: Axial view showing the reduction of the right nasal mass (red arrow). C, D: Axial view showing the reduction in cervical lymphadenopathy

An evaluation computed tomography scan after three cycles indicated a reduction of the right nasal mass to 20x12 mm from 57x16 mm, with persistent erosion of the nasal septum and medial wall of the maxillary sinus, and a reduction in cervical lymphadenopathy, the largest being in the right jugulo-carotid measuring 13 mm versus 30 mm (Figure 5).

After a multidisciplinary team discussion, the tumor was assessed as non-resectable, and the decision was made to proceed with concomitant chemoradiotherapy. After 10 weeks, the patient received concomitant chemoradiotherapy with a total dose of 59.4 Gray in 33 fractions of 1.8 Gray each. The concomitant chemotherapy was based on ifosfamide and vincristine.

The patient experienced grade 1 radiodermatitis and grade 2 pharyngeal radiomucositis. Post-therapeutic evaluation scan showed a complete response.

After 15 months of follow-up consultations, the patient presented with a multi-metastatic progression and passed away after three months.

## Discussion

Soft tissue sarcomas are unusual in adults, primarily affecting the pediatric population [5,6]. Rhabdomyosarcomas account for more than half of these cases in children [7]. In the adult population, this percentage experiences a notable decline, typically remaining within the range of 2-5% [8].

Understanding the pathogenesis of rhabdomyosarcoma in adults remains limited. Tumor progression has been attributed to mutations in the macrophage migration inhibitory factor (MIF) and p53 [5]. In contrast to pediatric cases, where clear risk factors, such as prenatal drug exposure and radiation exposure, are recognized, there is a notable absence of published studies investigating the origins of adult rhabdomyosarcoma [9].

In 1958, Horn et al. introduced a widely accepted classification system that includes embryonal, botryoid (a subtype of embryonal), alveolar [10], and pleomorphic types [7]. The World Health Organization (WHO) further categorizes this tumor into embryonal, alveolar, pleomorphic, and spindle cell/sclerosing rhabdomyosarcoma [5]. Diagnosis typically involves the application of an initial immunohistochemical panel, commonly including pan-cytokeratin, CD45RB, and protein S100 markers. Stout et al. were the first to categorize rhabdomyosarcoma tumors in 1946 [11].

Alveolar rhabdomyosarcoma constitutes 20-30% of rhabdomyosarcoma in patients aged 15 to 20 years [12]. It primarily originates from the deep soft tissues of the extremities, paraspinal regions, perineal areas, and paranasal sinuses [12]. Tumors occurring in the orbit, non-para-meningeal locations of the head and neck, and the male or female genitourinary systems are considered “favorable”. All other locations are considered "unfavorable": the bladder or prostate, an arm or leg, a parameningeal site (an area next to the membranes covering the brain, such as the nasal passages and nearby sinuses, middle ear, or the uppermost part of the throat), This is the case of our patient.

To our knowledge, only a few cases have been reported in the paranasal sinuses in adults. These tumors can be confused with other malignant soft tissue tumors [13].

The initial diagnosis of our patient is similar to what is reported in the literature. The difficulty in confirming the diagnosis and subsequent treatment delays are factors for poor prognosis in adult rhabdomyosarcoma. Initial nasal symptoms, such as nasal congestion, and ocular symptoms, such as progressive proptosis and tearing, should be included in the differential diagnosis to improve early diagnosis [14].

The most common staging systems are the TNM-UICC and Intergroup Rhabdomyosarcoma Study systems [12].

A comprehensive workup is therefore necessary, including a biological assessment, thoracoabdominal computed tomography scan, hepatic ultrasound, bone scintigraphy, and fluorodeoxyglucose positron emission tomography [15]. Magnetic resonance imaging and computed tomography are more suitable for visualizing anatomy. The value of fluorodeoxyglucose positron emission tomography in treatment evaluation is still unclear and needs to be explored in more detail. However, it has proven to be an excellent tool for assessing treatment efficacy in a special group of soft tissue sarcomas [15].

The standard approach for managing rhabdomyosarcoma typically involves a multidisciplinary approach, incorporating chemotherapy and surgical interventions, sometimes supplemented with radiotherapy. Nevertheless, defining a standardized treatment regimen for adult rhabdomyosarcoma remains challenging. In most instances of adult rhabdomyosarcoma, surgical resection is pursued alongside chemotherapy and, in some cases, radiotherapy, aligning with treatment approaches established for pediatric patients, albeit with variations based on individual clinician discretion [16]. Furthermore, prognostic determinants for rhabdomyosarcoma include factors such as patient age, tumor size, invasiveness, presence of metastases, regional lymph node involvement, and the tumor's pathological response to chemotherapy [17,18].

In general, adult rhabdomyosarcoma is managed with neoadjuvant chemotherapy associated with locoregional treatment. Chemotherapy protocols often combine vincristine, actinomycin, cyclophosphamide, etoposide, ifosfamide, and doxorubicin. Rhabdomyosarcoma tumors are radiosensitive, with a pediatric dose of 50.4 gray for embryonal and alveolar rhabdomyosarcomas [19]. With the advent of CT imaging, there has been a significant transition to three-dimensional planning, which has persisted and evolved to include advanced modalities that offer even greater dose conformality such as intensity-modulated radiation therapy (IMRT) and proton therapy.

Thanks to clinical trials and intensive treatment strategies, treatment outcomes for children and adolescents with rhabdomyosarcoma have significantly improved over the past decades [20]. However, the prognosis for rhabdomyosarcoma in adults remains poor [17]. A previous series showed a 5-year overall survival rate of 27% in adult rhabdomyosarcoma compared to 61% in pediatric rhabdomyosarcoma [18,21].

The poor prognosis associated with adult rhabdomyosarcoma stems from several factors. Initially, adult patients with rhabdomyosarcoma frequently present with advanced stages of the disease. A prior investigation indicates that over 60% of adults diagnosed with rhabdomyosarcoma exhibit regional or distant metastases upon initial diagnosis [22]. Additionally, adult rhabdomyosarcoma is characterized by a notable incidence of metastatic relapse [23], and decreased tolerance to treatment, leading to lower therapeutic doses [23,24]. Furthermore, adult rhabdomyosarcoma often manifests with histopathological subtypes or anatomical locations that are less favorable compared to pediatric counterparts [23,25], and more significantly, there is currently no established standard treatment approach for adult rhabdomyosarcoma [24,26]. Recent clinical trials targeting rhabdomyosarcoma have predominantly focused on pediatric and adolescent populations [27,28].

## Conclusions

Alveolar rhabdomyosarcoma presents a challenge in the medical field due to its uncommon occurrence. Timely diagnosis and aggressive treatment are essential for improving outcomes. Our case highlights the poor prognosis associated with alveolar rhabdomyosarcoma, characterized by a high risk of metastasis, as evidenced by the patient's post-therapy progression.
